# Cell-Derived Matrix: Production, Decellularization, and Application of Wound Repair

**DOI:** 10.1155/2024/7398473

**Published:** 2024-05-29

**Authors:** Yidan Xu, Yao Yao, Jianhua Gao

**Affiliations:** Department of Plastic and Cosmetic Surgery Nanfang Hospital Southern Medical University, 1838 Guangzhou North Road, Guangzhou 510515, Guangdong, China

## Abstract

Chronic nonhealing wounds significantly reduce patients' quality of life and are a major burden on healthcare systems. Over the past few decades, tissue engineering materials have emerged as a viable option for wound healing, with cell-derived extracellular matrix (CDM) showing remarkable results. The CDM's compatibility and resemblance to the natural tissue microenvironment confer distinct advantages to tissue-engineered scaffolds in wound repair. This review summarizes the current processes for CDM preparation, various cell decellularization protocols, and common characterization methods. Furthermore, it discusses the applications of CDM in wound healing, including skin defect and wound repair, angiogenesis, and engineered vessels, and offers perspectives on future developments.

## 1. Introduction

At the macroscopic clinical level, the principles of treating chronic wounds include debridement, maintaining moisture balance in the wound microenvironment, preventing infection, and treating systemic diseases affecting the wound. These principles significantly reduce inflammation and excessive proliferation in wound healing [1]. Concurrently, chronic wound physiology has proven highly complex at the cellular level, involving numerous regulatory axes and signaling pathways [2, 3, 4]. The wound healing research has focused on cytokines like epidermal growth factor, fibroblast growth factor, and transforming growth factor 1. Increasingly, wound care products are targeting the “microscopic” environment of the wound, leading to customized therapeutic materials. Currently, widely used wound care materials aim to alleviate symptoms such as exudate, infection, and wound tension. In contrast, developing biomaterials based on mimicking the extracellular matrix not only alleviates these symptoms but also modulates immune responses to effectively reduce inflammation [5, 6, 7]. Decellularized matrix natural biomaterials obtained through decellularization techniques have emerged as an alternative strategy for developing wound healing materials, increasingly attracting attention in tissue engineering research. Components of the ECM and the decellularized matrix itself have become promising scaffolds in tissue engineering. The ECM is a complex 3D network composed of biomacromolecules, present in nearly all connective tissues as an acellular structure. It primarily consists of two major categories of substances: fibrous proteins (fibronectin, laminin, and collagen) and proteoglycans (glycoproteins, polysaccharides, etc.). Research has shown that these molecules not only provide structural support throughout the cell life cycle but also influence cellular morphology, metabolism, secretion, and migration processes [8, 9, 10, 11, 12]. Given the significant role of the ECM, researchers have developed natural decellularized ECM as scaffolding materials for regenerative medicine. These scaffolds, derived from animal organs or tissues, retain some natural microstructures and bioactive factors [13, 14]. Studies have shown that decellularized ECM materials effectively regulate stem cell proliferation, maintain stem cell phenotypes, and promote differentiation [13, 15]. They contain an abundance of natural bioactive factors (VEGF, TGF-*β*, IGF-1, HGF, FGF-2, etc.) and immunomodulatory factors (TNF-*α*, IL-11, IL-7, IL-10, IL-8, IL-6, etc.) [16, 17], which have demonstrated significant healing acceleration in animal wound models, thus presenting promising prospects in skin tissue engineering. However, challenges such as donor shortage, the destructive impact of robust decellularization methods on the ECM's natural microstructure, variations between decellularized tissue batches, and potential risks of pathogen transmission have limited the development of organ/tissue-derived decellularized ECM in tissue engineering [8, 18, 19, 20, 21, 22].

Cell-derived extracellular matrix (CDM) serves as an alternative to organ/tissue-derived ECM, relying on in vitro cell culture techniques to harvest short-term ECM secretions from cells and then decellularize them. This approach offers more flexible strategies for tissue engineering design [23, 24]. CDM addresses the limitations associated with organ/tissue-derived ECM by facilitating continuous ECM production in vitro, thus overcoming issues related to donor scarcity; it also allows for batch-to-batch consistency and biosafety through selective cell culture screening [21, 25, 26]. Furthermore, CDM exhibits unique advantages, such as excellent biocompatibility and operability [27, 28, 29]. Its simpler decellularization process results in better preservation of glycosaminoglycans (GAGs) in the ECM and a higher proportion of basement membrane proteins being secreted. The increased levels of GAG and basement membrane proteins, such as type IV collagen and laminin, positively influence regenerative activities like proliferation and angiogenesis [30]. CDM production is sustainable, reducing the use of experimental animals and making it a practical biomaterial in tissue engineering.

In biomedical research, tissue engineering, and regenerative medicine, CDM is extensively used. In biomedical research, CDM provides a platform to study the tumor cell microenvironment [31] and explore the mechanisms of cell-ECM interactions [32, 33, 34]. In tissue engineering, CDM is used to design 3D bioprints, as coatings for material surfaces, and in cross-linking with other materials [35, 36, 37]. The use of CDM in transplant surgeries for repairing tissue defects, such as in orthopedics, dermatology, and ophthalmology, has shown positive outcomes [38, 39, 40, 41, 42, 43]. CDM can also serve as a supportive material for stem cell therapies. Stem cells cultured on CDM demonstrate enhanced survival rates and biological activity. Studies have shown that using CDM as a scaffold for MSCs increases osteogenic potential compared to *β*-tricalcium phosphate (*β*-TCP) and promotes higher expression of osteogenesis-related factors like VEGF and BMP-2 [44, 45]. Furthermore, CDM has been shown to modulate cell behaviors. For instance, Zhang et al. [46] explored the effects of growth and adipogenic media on the ECM secretion profiles of ADSCs cultured on CDM. Their findings indicated that ADSCs grown in growth media on CDM exhibited increased migratory capabilities, whereas those grown in adipogenic media underwent adipogenic differentiation. Similar studies have investigated the effects of CDM secreted by C2C12 myoblasts on stem cell myogenesis [47].

This article reviews the current preparation processes of CDM, including cell expansion, decellularization protocols, and characterization strategies, while introducing some innovative design concepts. Additionally, CDM has demonstrated excellent effects in wound healing and angiogenesis. The paper summarizes the applications of CDM in skin tissue engineering and offers perspectives on future developments.

### 1.1. The Production Process of CDM

**Figure 1:**
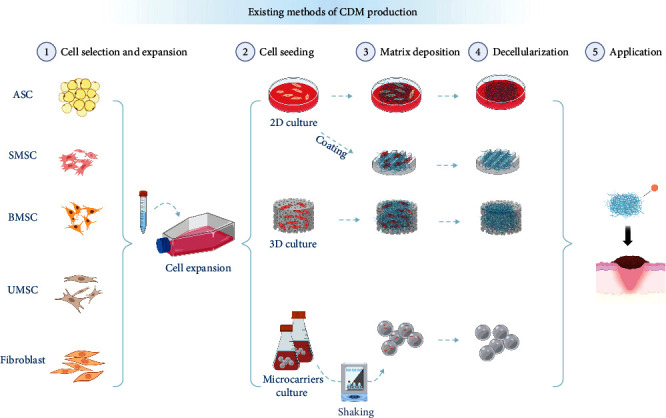
Existing methods of CDM production. The process consists of four main steps: cell selection and expansion, cell seeding, matrix deposition, and decellularization. Cell selection and expansion: cell types should be selected and then expanded to sufficient numbers under in vitro cell culture techniques. Cell seeding: cells are inoculated on 2D planes or prepared as coated materials or combined with 3D scaffold materials. 3D cell sphere preparation requires shaking floating cultures. Matrix deposition: matrix deposition relies on artificially added chemicals, with the most basic additive being ascorbic acid. Creating an environment of macromolecular crowding can also promote matrix deposition. Decellularization: the CDM decellularization method is simple and has low chemical residues.

The preparation process of CDM varies depending on application requirements and includes several design options. It primarily comprises four stages (Figure 1): cell selection and expansion, cell seeding, matrix deposition, and decellularization [48].

### 1.2. Cell Selection and Expansion

Prior to cell expansion, the choice of cell types for in vitro culture should align with the intended application. CDMs harvested from different primary cultured cells exhibit subtle structural and molecular composition differences, resulting in distinct functional focuses. For example, while CDMs derived from bone marrow stromal cells (BMSCs) contain fewer angiogenic factors compared to those from human umbilical cord mesenchymal stem cells, they significantly enhance the reconstruction of accessory organs during wound healing. CDMs from adipose-derived stem cells (ASCs) not only promote angiogenesis but also exert significant immunomodulatory effects. Fibroblast-derived CDMs, with their richer protein deposition, offer superior mechanical strength than other CDMs, making them more promising for designing various wound care adjuncts [8, 32, 38, 39, 49, 50].

Once the cell type is determined, the process should begin with the acquisition, isolation, identification, and either cryopreservation or direct expansion of the primary cells. Extensive expansion of the target cells is necessary to ensure that the quantity of matrix deposited meets experimental requirements, making the acquisition of a sufficient number of cells a critical step in the cell expansion phase. Additionally, it is crucial to maintain cell viability and minimize cell senescence during expansion, as these factors significantly affect subsequent matrix acquisition [51].

### 1.3. Cell Seeding

Based on application needs, there are three general cultivation methods for expanded cells: culture on 2D surfaces (dish culture/material coating), in 3D materials, and as 3D cell spheroids. 2D culture is characterized by its simplicity and ease of decellularization for matrix deposition [24, 27]. When cultured in 3D materials, the ECM obtained more closely mimics the microstructure of natural tissues. Additionally, 3D culturing allows for the integration of CDM with biodegradable materials to construct specific geometric shapes tailored to the application. For 3D spheroid cultures, cell expansion is followed by formation using self-assembly or agitation methods. Microcarriers, which provide a larger growth surface area than 2D cultures, are commonly used to expand adherent cells. Generally, these are porous or solid microspheres, which can be surface-modified and functionalized according to cell type. They are used with stirred-tank bioreactors to expand MSCs, pluripotent stem cells, and embryonic stem cells [52, 53]. 3D culturing techniques generate ECM that more accurately mimics natural microstructures. While cells cultured in spheroids exhibit a slight decrease in viability and proliferation, they demonstrate increased expression of transcription factors related to stemness. This 3D culture method enhances mitochondrial DNA content, oxygen consumption rate, and extracellular acidification rate, while also increasing the production of reactive oxygen species and reducing lactate levels within the cells. Moreover, the macromolecular crowding environment induced by the spheroids facilitates the rapid deposition of ECM [23, 54, 55, 56, 57, 58].

### 1.4. Matrix Deposition

Matrix deposition is a crucial step in preparing CDM, as it determines the production of CDM necessary for experiments. To expedite ECM deposition, which naturally occurs slowly over the cellular growth cycle, researchers often need several weeks of culture with large cell volumes. This limitation can be improved by modifying the cultural environment [59, 60]. Continuous strategic improvements are essential; for instance, ascorbic acid is a fundamental additive that serves as a cofactor for lysyl and prolyl hydroxylases, effectively enhancing collagen fiber deposition and thus increasing the overall yield of CDM. Similarly, agents like ficoll, carrageenan, or dextran sulfate can be used to simulate the in vivo macromolecular crowding environment in 3D spheroid cultures to accelerate matrix deposition [54, 61, 62, 63, 64]. Hypoxic conditions have been shown to induce higher levels of vascular growth factors and collagen in CDM [65, 66, 67], while inhibiting metalloproteinases in the culture have been proposed to reduce ECM degradation by lowering serum concentrations [68, 69]. However, both conditions may negatively affect cell growth, requiring further evidence to confirm that these in vitro environments positively influence in vivo material tests.

Besides altering external factors, genetic modification techniques can also be employed to engineer stem cells [70], producing CDM materials with desired physical and biochemical properties tailored to specific applications.

### 1.5. Decellularization

**Figure 2:**
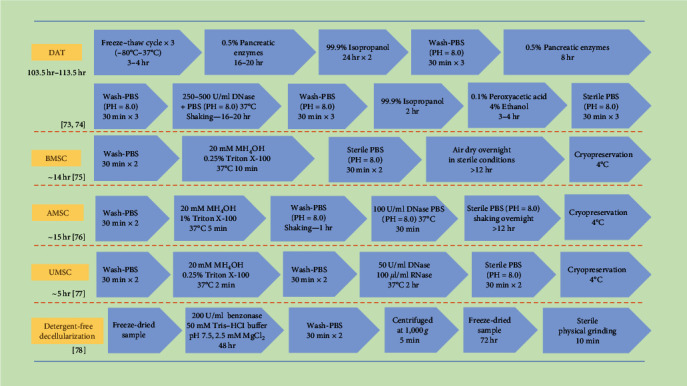
Decellularization process of various mesenchymal stem cells and comparison with scaffold materials of decellularized adipose tissue. DAT, decellularized adipose tissue; AMSC, adipose mesenchymal stem cells; BMSC, bone marrow mesenchymal stem cells; UMSC, umbilical vein mesenchymal stem cells.

The decellularization process involves the use of physical, chemical, and enzymatic methods to remove cellular components, which is critical for reducing the immunogenicity of materials [71, 72]. However, during decellularization, the biomaterial's bioactive factors and 3D microstructures are often compromised. Thus, balancing the extent of decellularization with the preservation of bioactive factors is a considerable challenge. Here, we summarize the commonly used decellularization protocols for CDM (Figure 2) and compare them with those used for traditional adipose tissue-derived ECM. Due to the typically smaller scale of CDM preparations, it is possible to employ gentler decellularization methods, not only simplifying the process but also better preserving the structure and bioactive factors [30, 79, 80, 81].

Physical and mechanical decellularization methods include freeze–drying, ultrasonication, or pressurized freeze–drying [14, 82]. Freeze–thaw cycles can disrupt cell membranes, releasing intracellular contents and further removing cellular components. While physical decellularization methods can effectively preserve the biochemical composition of the ECM, such as GAGs, collagen, TGF-*β*, VEGF, and IGF-1, they substantially alter the mechanical properties and conformation of collagen. Changes in collagen conformation can significantly affect the biological functions of the ECM [75, 83, 84].

Chemical decellularization techniques employ agents like Triton X-100, sodium deoxycholate (SDC), and sodium dodecyl sulfate (SDS) to effectively eliminate DNA while also altering the biochemical properties of the ECM [14, 73]. Triton X-100, a nonionic surfactant, preserves bioactive factors and maintains the structural integrity of ECM fibrous proteins. Research indicates that Triton X-100 successfully sustains the functionality of bioactive compounds. The production of these proteins involves numerous elements, such as platelet-derived growth factors, bone morphogenetic protein-4, epidermal growth factors, fibroblast growth factors, hepatocyte growth factors, and vascular endothelial growth factors. However, it does decrease the GAG levels in CDM and alters some collagen structures [85, 86, 87]. As ionic detergents, SDC and SDS significantly denature ECM proteins, reducing the CDM's affinity for binding factors like VEGF and TGF-*β*. Furthermore, chemical decellularization processes also incorporate acidic and alkaline solutions, along with hypo- and hypertonic solutions, which not only strip away cellular elements but also deplete growth factors and modify the ECM [20, 74, 88].

Enzymatic decellularization methods include the use of DNases and RNases, which remove genetic materials without impacting proteins [78]. A thorough decellularization often involves a blend of these techniques. Traditional approaches involve repeatedly freezing and thawing organs and tissues, followed by chemical decellularization of the ECM, making it a protracted and intricate process. As a result, this extends processing times and significantly changes the ECM's morphology [89]. Additionally, stringent washing protocols are necessary to ensure biological safety. In contrast, the combined decellularization of CDM is simpler and quicker. Given the brief production cycle of CDM, it is more prone to losses during decellularization and washing compared to tissue-derived ECM. Moreover, the safety concerns associated with residual chemicals in decellularized CDM warrant further investigation [90]. Despite the development of purely physical decellularization methods, concerns about the risks posed by chemical agents persist. To compensate for reduced yields, the current strategy involves ramping up ECM production initially. The goal of decellularization is to minimize immunogenicity, with immunomodulatory strategies emerging as promising new avenues. Research by Liu et al. [91] demonstrates that acellular adipose matrices modified with mPEG result in reduced antigenic exposure, enhanced tissue regeneration, and better graft retention in xenotransplant scenarios.

### 1.6. Characterization of CDM

After obtaining CDM, it must be characterized to confirm that it meets experimental expectations. Common characterization methods for CDM are outlined in Table 1. First, the effectiveness of decellularization is verified. This can be approximated by hematoxylin and eosin staining to observe the presence of cellular nuclei or by using nuclear fluorescent staining. Similarly, DNA quantification can assess residual DNA postdecellularization, with results expected to be less than 50 ng/mg [78]. Subsequently, a range of techniques can be used to analyze the biochemical composition and physical properties of the obtained CDM. These include Western blotting to measure specific protein content, hydroxyproline assays to determine total collagen content, or proteomics techniques for a comprehensive analysis of the CDM. Immunofluorescence staining can be used for the localization and distribution of specific molecules. Structurally, the microstructure of CDM can be observed using scanning electron microscopy (SEM) and transmission electron microscopy (TEM). Additionally, mechanical properties such as Young's modulus can be measured using a mechanical testing device [24, 49, 93, 94, 95, 96, 97, 99].

### 1.7. Application of Wound Repair

**Figure 3:**
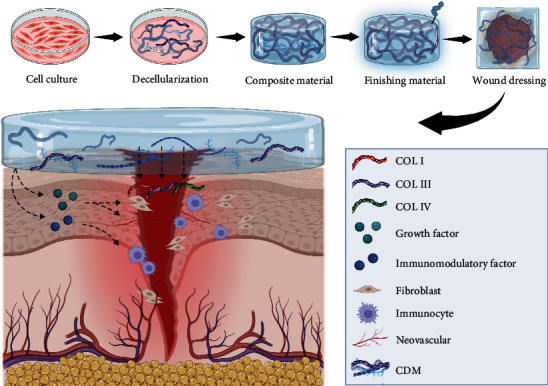
Application of CDM in skin wound repair. The CDM is prepared through a series of processes, including cell expansion, ECM deposition, decellularization, and modification. Once prepared, the CDM serves as a dressing for skin wounds. Rich in collagen, the CDM facilitates wound healing by promoting fibroblast proliferation at the wound site, which aids in tissue regeneration. Additionally, CDM releases bioactive factors that regulate immune responses, notably by modulating macrophage activity to reduce inflammation. The growth factors secreted by the CDM also enhance angiogenesis and neurogenesis, further supporting the wound-healing process.

Suitable dressings play a significant role in promoting the repair and healing of skin wounds. Over the past few decades, tissue engineering dressings have become a viable option for treating chronic, hard-to-heal wounds. Among these, CDM exhibits excellent biocompatibility and high concentrations of growth factors, making them particularly effective in aiding skin regeneration and healing [70, 71, 100] (Figure 3). Decellularized extracellular matrix materials derived from various types of mesenchymal stem cells have been reported to positively impact wound repair, each type of cell contributing uniquely due to differences in physical properties or biochemical composition. Additionally, due to the processability of CDM, the collected material can be fabricated into hydrogel patches or injectable hydrogels [90, 101].

Adipose-derived mesenchymal stem cell-derived matrix (ASC-CDM) has shown significant potential in promoting wound healing, garnering attention in the field of skin tissue engineering. Previous studies have demonstrated that ASC-CDM enhances wound healing and skin regeneration through immunomodulation, vascular regeneration, support of fibroblast proliferation, and reduction of tissue scarring [67, 102, 103]. ASC-CDM exhibits increased deposition of collagen types I, III, and IV, along with soluble growth factors such as VEGF and TGF-*β*, which are essential for wound healing. When applied to wound models, ASC-CDM mediates cellular behavior through integrin-mediated signaling pathways on cell surfaces and regulates fibroblasts and keratinocyte actions, thus promoting wound closure [104, 105]. BMSCs have demonstrated effective wound repair in vivo experiments with nude mice, with the application of BMSC-CDM notably enhancing the reconstruction of accessory structures and vascular functionality [38, 49]. Human umbilical cord blood-derived mesenchymal stem cells have shown superior wound healing and skin regeneration in comparative studies with other cultured cells used for wound repair, likely due to their efficient recruitment of M2 macrophages and significant pro-angiogenic effects [50]. Mesenchymal stem cells, compared to terminally differentiated fibroblasts, exhibit greater synthesis abilities for extracellular matrix proteins and developmental proteins. Proteomic analysis of their CDM has revealed significant upregulation of fibronectin and ECM proteins, suggesting that MSC-derived CDM may facilitate wound healing through support of integrin-mediated cell adhesion, signal transduction, and migration [106, 107].

Fibroblast-derived CDM promotes wound healing and angiogenesis. When designing skin wound healing materials using fibroblasts, the type of fibroblast is a critical design consideration [39, 108, 109]. In vitro experiments reveal that CDMs from pulmonary fibroblasts, dermal papilla fibroblasts, and reticular fibroblasts each have distinct morphologies and characteristics. While all three types of CDM support wound healing, pulmonary fibroblasts produce a richer array of vascular growth factors and tend to promote macrophage polarization toward the M2 phenotype. Dermal papilla fibroblasts are particularly effective at inducing the formation of epidermal basement membrane-like structures, likely due to their tissue origin [50, 110]. Research by Santarella et al. [111] using pluripotent stem cell-derived fibroblasts shows that their CDM contains a richer array of structural proteins compared to CDM from human fibroblasts.

Effective vascularization is crucial for wound repair. CDM has demonstrated significant angiogenic capabilities, confirmed by the observed activation of endothelial cells in wound models [77]. Previous studies have cocultured adipose stem cells with microvascular endothelial cells, observing proactive self-assembly of prevascular structures in vitro, and confirmed similar effects with adipose-derived CDM [76, 112]. The use of human umbilical cord blood mesenchymal stem cells and umbilical vein endothelial cells has induced enhanced vascular formation [113]. In cases of significant vascular deficits in local tissues, vascular smooth muscle cells, endothelial cells, and fibroblasts can be seeded on 3D scaffolds to deposit ECM and construct vessel-like structures [114, 115].

The release kinetics of bioactive factors within CDM should be considered during material design. Different factors vary in their release efficiency when applied to wounds. Research by Greenwood-Goodwin et al. [116] found that smaller molecular weight bioactive factors release more quickly over extended periods, while no significant differences are observed in the short term (within 7 days). Decellularized dermal matrices are already used clinically, including products such as Dermagraft®, Apligraft®, Allopatch®, GraftJacket®, and Integra®. These products, popular as decellularized tissue transplants, are derived from whole tissues through decellularization [117]. CDM, due to its straightforward decellularization protocol and processing flexibility, can be prepared as decellularized ECM powder, hydrogels, and composite grafts. Decellularized CDM is ground into a powder after freezing and lyophilization and stored frozen. This powdered form of CDM can flexibly cover tissue defects at wound sites, with strong reprocessability into hydrogels and bioprinting inks for injectable wound treatments. Additionally, CDM powder serves as a supportive material for cell therapies, enhancing cell survival and function in the host [118, 119]. When converted into a hydrogel, CDM broadens its potential applications both in vitro and in vivo. ECM-based hydrogels mimic the physiological matrix environment, facilitating cell adhesion, infiltration, and proliferation. The decellularized CDM retains type I collagen and laminin, is hydrophilic, and possesses the mechanical strength that is suitable for wound healing. Despite the promising prospects of CDM, it is challenging to replicate the structure, composition, and mechanical properties of natural CDM [120]. Thus, to produce CDM with relevant mechanical properties, scaffolds incorporating biodegradable polymers have been developed, such as those using PLGA electrospun nanofiber templates with ASC for wound healing dressings [121] or biphasic calcium phosphate scaffolds enriched with ECM derived from rat BMSCs for mechanical and biological functionalization of scaffolds [29]. These scaffolds show increased osteoblastic differentiation and upregulation of osteoblastic genes [117, 122]. Compared to artificial materials, CDM provides a more natural and cell-compatible microenvironment and contains bioactive factor concentrations several times higher than traditional methods [71], making it an ideal dressing for chronic wounds. However, the production of CDM is still limited by traditional cell culture techniques, and its efficiency is insufficient to meet clinical demands. Industrial-scale production remains challenging in the short term, which may be the biggest obstacle CDM faces.

## 2. Conclusion

Over recent decades, CDM has progressively advanced within tissue engineering, with many studies focusing on the repair of both in vivo and in vitro tissue defects. This includes regeneration of skin wounds, bone and cartilage, peripheral nerves, and periodontal tissues. Due to its microenvironment and biochemical composition similar to natural ECM, along with its straightforward procurement, CDM has become an ideal scaffold material in tissue engineering. Researchers can use CDM to construct surface coatings, mesh materials, and hydrogels that closely mimic the natural tissue environment, and they can also prepare them as delivery vehicles for cell transplantation. CDM is a valuable and widely applicable biomaterial with potential applications ranging from basic medical research to clinical regenerative medicine. It can be produced in a controlled laboratory setting to simulate physiological environments based on specific needs. As a biomaterial, CDM is easily sourced and eliminates the use of experimental animals, allowing for sustainable in vitro production. It also offers high adjustability in terms of parameters, and its modification and regulation are relatively straightforward. However, research related to CDM still faces limitations.

First, there is no standardized protocol for the preparation of CDM, including cell sourcing, selection, preparation, storage, and quality control. Additionally, the efficiency of CDM deposition has historically hindered research progress. Low production efficiency and the lack of uniform production standards have delayed clinical applications. As a biomaterial, CDM inevitably faces biosafety issues, necessitating more stringent selection schemes and characterization methods. Although numerous design proposals for CDM preparation have been suggested, the protocols applied in practice remain relatively uniform, likely due to ongoing challenges in modulating cell and ECM production.

It is noteworthy that with the advancement of the biomaterials field, CDM is expected to integrate with an increasing variety of material production techniques, including 3D printing and enhancements in cellular niches within organ-on-chip systems [123, 124]. CDM materials with controllable preparation parameters are also becoming vital tools in physiology, pharmacology, and disease modeling due to their excellent simulation of physiological and pathological environments. In summary, CDM has not yet fully demonstrated its biological potential. There is anticipation that future research breakthroughs in preparation methodologies will significantly enhance the prospects for CDM.

## Figures and Tables

**Table 1:** Common characterization of CDM.

Categories	Characterizations	Presence to verified	Ref.
DNA quantification	DNA quantification kits (e.g., Picgreen)	Residue genetic material	[78, 92]

Biochemical analyses	Hydroxyproline assay	Total collagen content	[24, 93]
	GAG assay	Total glycosaminoglycans content	[71, 93]
	Western blot enzyme-linked immunosorbent assay	Specific components in ECM	[30, 94, 95, 96]

Imaging techniques	Immunostaining	Position and morphology of target molecules	[30, 97]

Proteomic analysis	Mass spectrometry	Components in ECMs	[49, 98]

Optical detection	Atomic force microscopy (AFM)	Surface roughness	—
	Scanning electron microscopy (SEM)	Sectional and surface structure	—
	Transmission electron microscopy (TEM)	Assembly of collagen fiber arrangement	[38, 50, 99]

The commonly used CDM characterization methods are summarized, and the application purposes are listed. CDM, cell-derived matrix; ECM, extracellular matrix; GAG, glycosaminoglycan.

## Data Availability

The data underlying this article will be shared upon reasonable request to the corresponding author.

## Additional Points

*Impact Statement*. The CDM is a decellularized extracellular matrix (ECM) obtained through laboratory culture processes. Due to its favorable biological properties, CDM has garnered sustained attention in the field of tissue engineering. CDM scaffolds reduce the immunogenic risks associated with xenografts of traditional decellularized matrices, lower the risk of pathogen transmission, and decrease the use of experimental animals. Additionally, their enrichment with growth factors and basement membrane proteins positively impacts wound repair. This article reviews the existing preparation processes of CDM materials and their applications in wound healing and angiogenesis, providing readers with insights into CDM preparation schemes and related applications.
